# Balancing Knowledge and Health: A Comparative Analysis of Students and Healthcare Workers Nutrition Related Health Behaviors, a Cross‐Sectional Study

**DOI:** 10.1111/nhs.70000

**Published:** 2024-11-18

**Authors:** Hussein Zaitoon, Lisa Kaly, Hadel Khalil, Nataly Zion

**Affiliations:** ^1^ The Institute of Pediatric Endocrinology, Diabetes and Metabolism, Dana‐Dwek Children's Hospital, Tel Aviv Sourasky Medical Center, Affiliated to the Faculty of Medicine Tel Aviv University Tel Aviv Israel; ^2^ Rheumatology Unit, Bnai Zion Medical Center Faculty of Medicine Haifa Israel; ^3^ Galilee Medical Center Nahariya Israel; ^4^ Bnai Zion Academic School of Nursing Haifa Israel

**Keywords:** healthcare workers, healthy lifestyle, Mediterranean diet score, nurses, nutrition assessment, public health

## Abstract

The study aimed to investigate nutrition‐related health behaviors among nursing and medical students compared to healthcare workers (HCWs), including nurses and physicians. A cross‐sectional survey was conducted between May and November 2022, using the I‐MEDAS and lifestyle‐related behavior questionnaires. The participants included nursing and medical students, nurses, and physicians, with a total of 384 participants: 16.9% physicians, 23.17% nurses, and 59.9% students, 93% of whom were nursing students. The average I‐MEDAS score (out of 17) was 7 for physicians, 8 for nurses, and 8 for students, with no significant differences in lifestyle behavior scores between the groups (*p* = 0.11), although nurses tended to score lower. Nurses also had a significantly higher BMI compared to physicians and students (*p* < 0.001). Physicians and nurses reported lower engagement in leisure activities and household chores, fewer sleep hours, and poorer sleep quality. The findings highlight a decline in healthy lifestyle behaviors and poor adherence to the Mediterranean diet among HCWs and students, particularly nurses, emphasizing the need for interventions to promote healthier habits in these groups.

**Summary**

- This study demonstrates the overall negative change in lifestyle behaviors and suboptimal adherence to Mediterranean diet among nursing and medical students, nurses, and physicians, highlighting no gender differences.
- The study identifies specific challenges faced by nurses and physicians, including shift related food choices and weight gain, emphasizing the need for targeted support.
- This paper emphasizes the importance of ongoing support and resources for healthcare workers to sustain their health, setting an example for patients and contributing to the overall well‐being of healthcare professionals and the healthcare system.

## 1 Introduction

Numerous studies have established a correlation between an unhealthy diet and various ailments such as obesity, diabetes, heart disease, and cancer (Cena and Calder 2020). However, adopting a healthy lifestyle that includes a nutritious diet has been proven to prevent these conditions. Eating behavior pertains to one's eating habits, food preferences, portion sizes, and food preparation methods. Sociocultural, environmental, and genetic factors significantly influence eating behavior (Emilien and Hollis 2017). Any deviations from a balanced eating behavior can have a profound impact on an individual's health.

The foundation of a healthy diet is primarily composed of vegetables, fruits, legumes, whole grains, dairy products, and plant‐based fats. Studies have shown that by promoting cardiovascular health and metabolic function, the Mediterranean diet supports longevity and reduces the risk of chronic diseases, such as heart disease, stroke, diabetes, and certain types of cancer that can lead to premature death (Dominguez et al. 2021; Tuttolomondo et al. 2019). The Mediterranean diet emphasizes a high intake of plant‐based foods such as vegetables, fruits, legumes, and nuts, with olive oil serving as the primary source of fat. Additionally, it suggests minimizing the consumption of red and processed meat while favoring eggs, chicken, and fish. The World Health Organization and the Israeli Ministry of Health have both incorporated the principles of the Mediterranean diet into their official recommendations (World Health Organization n.d.; Ministry of Health n.d.).

Healthcare workers (HCWs), particularly those working in hospitalization settings, frequently work shifts to provide 24‐h service, which has an effect on their meal choice and timing. The abnormal timing of meals due to shift work can have adverse effects on the digestion process, nutrient absorption, enzyme activity, hunger and satiety sensations (Amani and Gill 2013; Lowden et al. 2010). Shift work may also negatively impact eating behavior, causing individuals to eat at night, have fewer main meals, lower diet quality, and consume more calories, fatty acids, and coffee (Buchvold et al. 2015). Moreover, several studies have shown that shift workers have a high risk of chronic diseases such as metabolic syndrome and obesity (Zhang et al. 2020), cardiovascular diseases (Torquati et al. 2018), diabetes (Gao et al. 2020), and sleep disorders (Pallesen et al. 2021).

In addition to the challenges faced by healthcare workers, poor nutritional behaviors also have significant health implications for students, particularly medical and nursing students. While these students may have greater knowledge of healthy lifestyle practices due to their education, the intense demands of their academic programs, long study hours, and irregular schedules often lead to poor dietary habits (Thwaite et al. 2020). This can lead to skipped meals, increased snacking, and reliance on fast, unhealthy foods, potentially contributing to long‐term issues like obesity, metabolic syndrome, and cardiovascular problems (Mkanzi et al. 2020; Romero‐Blanco et al. 2022). The stress and pressure of medical and nursing education can further exacerbate unhealthy eating habits, leading to a vicious cycle of poor diet, stress, and adverse health outcomes (Kritsotakis et al. 2020; Macedo et al. 2020). Thus, the health implications of poor nutritional behaviors among students are equally critical to address, as they are at risk of developing unhealthy patterns that can affect their well‐being throughout their lives. Despite the well‐known heavy workload and emotional stress that come with their chosen profession, the curriculum of medical and nursing schools does not adequately prepare students for lifestyle changes, particularly in their diets (Thwaite et al. 2020).

The primary objective of this study is to identify, via an anonymous questionnaire, the changes in lifestyle patterns and unhealthy eating habits among nursing and medical students compared to health care professionals.

## 2 Material and Methods

### 2.1 Study Design

An anonymous cross‐sectional survey was administered via Google Forms to HCWs in North of Israel, including nursing and medical students, registered nurses, and hospital physicians. The survey was conducted from May 1, 2022, to November 1, 2022.

### 2.2 Study Population

The study included nursing and medical students from Zeide school of nursing and Technion medical faculty both located in Haifa city, northern Israel. Furthermore, registered hospital setting working nurses and physicians were included. Other paramedical staff were not eligible to participate.

To determine the necessary sample size for this survey study, we aimed for 80% power at a 95% confidence level to detect a medium effect size of 0.5 in adherence scores, based on responses to a 37‐item questionnaire. Assuming a standard deviation of 1.2, a minimum sample of 91 participants was required for robust statistical analysis of adherence and perception scores across the study population.

### 2.3 Data Collection

The survey was distributed via email or phone message, allowing participants to complete it only once. The authors obtained access to the institutional mailing and phone list of healthcare workers and students affiliated with Bnai Zion Medical Center and Zeide School of Nursing, ensuring that all contacted individuals were relevant to the study, access was provided with institutional approval. Participants were notified twice by email and once by phone message, ensuring that all relevant individuals were reached and given the opportunity to participate. Confidentiality of responses was clearly communicated to all participants. Respondents were encouraged to complete the entire survey, although it was not mandatory.

### 2.4 Ethical Considerations

The study adhered to the Equator and STROBE guidelines for reporting. Approval for the study was granted by the institutional review board Bnai Zion Medical Center under the approval number 0054‐22‐BNZ. The survey began with an explanation of its purpose, emphasizing the value of participation while clarifying that it was voluntary and not mandatory. Participants reviewed a consent statement, affirming their understanding and agreeing to participate before proceeding. The survey was anonymous to protect the privacy of the respondents.

### 2.5 Tools and Instruments

The survey initially covered participants' demographic and professional characteristics including age, gender, seniority, weight in kilograms at the time of filling the questionnaire and at the beginning of their studies or work period, current height, weekly hours of physical exercise, marital status including children number, current job status, and number of monthly night shifts. The subsequent items comprised two anonymous questionnaires.

The first part, the I‐MEDAS questionnaire, a 17‐item adapted Israeli Mediterranean Diet screener that demonstrated predictive utility for mortality in an adult Israeli population (Abu‐Saad et al. 2019). This tool is based on the Spanish PREDIMED study that led to 14 question tool to assess the dietary intake “Mediterranean Diet Adherence Screener” (MEDAS) which has been extensively explored as a tool to evaluate Mediterranean Diet adherence in various countries (Zazpe et al. 2010). The I‐MEDAS questionnaire assesses weekly food consumption and adherence to the principles of the Mediterranean diet. A scoring system was utilized for the survey, where one point was assigned for meeting each criterion and zero points for not meeting it. If the frequency of consumed portions was lower than indicated, the relative amount was not calculated, and zero was specified in the number of portions. For instance, an individual who consumes less than one serving of vegetables per day would indicate zero in the daily amount of vegetables. The final score ranged from 0 to 17 points, with a higher number of points indicating better adherence to the Mediterranean diet.

The validation of the I‐MEDAS was evaluated by (Abu‐Saad et al. 2019). The study evaluated both the content validity and the predictive utility of the I‐MEDAS within the Israeli population. The cohort was a representative, population‐based sample drawn that shares similar demographic and socio‐economic characteristics with the broader population. The study recruited 1318 individuals, with 1104 consenting to participate, achieving an 84% response rate. The participants were stratified by gender, ethnicity, and age group. To develop the I‐MEDAS, a professional committee of nutritionists and researchers adapted the original 14‐MEDAS to reflect the local Mediterranean diet and national dietary guidelines, resulting in a 17‐item questionnaire. The validation analysis demonstrated that the I‐MEDAS score, calculated based on food frequency questionnaire data, had strong predictive utility for mortality outcomes. Specifically, each 1‐point increase in the I‐MEDAS score was associated with a 12% reduction in the risk of all‐cause mortality (adjusted HR: 0.88; 95% CI: 0.80–0.97) (Abu‐Saad et al. 2019). In contrast, the original MEDAS score was less predictive of mortality and lost significance after adjusting for confounding variables (Abu‐Saad et al. 2019).

The second part of the survey comprised 20 questions, the lifestyle related behavior questionnaire; adapted from an Indian questionnaire designed to evaluate lifestyle changes during the COVID‐19 pandemic (Kumari et al. 2020), which was validated on 103 individuals older than 18 years with sampling adequacy of 0.688 established by Kaiser‐Meyer‐Olkin and the Bartlett test of sphericity (Chi‐squared, df = 190; *p*‐value < 0.001), with reliability assisted by Cronbach's alpha of 0.72. The questions were modified to suit the needs of healthcare workers (HCWs) and consent was obtained from the original authors for their use in this study and was assessed again for reliability with Cronbach's alpha coefficient of 0.82 (CI: 0.781–0.846) that suggests good internal consistency. The questionnaire comprises 20 items that comprehensively cover essential information for evaluating dietary habits (including intake, meal patterns, and snack consumption), physical activity (considering duration and type), and sleep (assessing both duration and quality). The diet‐related section [1–14] evaluates aspects such as the consumption of main meals, snacking habits, and the intake of both healthy (e.g., whole grains, fruits, vegetables, eggs, nuts) and unhealthy (e.g., fried food, junk food, sugar‐sweetened products) food items. Additionally, specific items address the intake of immunity‐boosting foods such as lemon, turmeric, garlic, citrus fruits and green leafy vegetables. The questionnaire also includes items [15–17] dedicated to assessing physical activity, including participation in aerobic exercise, household‐related activities, sitting time, and screen time. Furthermore, questions are included to determine sleep duration and quality. The scoring for this questionnaire was based on a 5‐point Likert scale, ranging from 1 to 5; with (1) significantly increased (2) slightly increased (3) grossly similar (4) slightly decreased and (5) significantly decreased. The scoring system included 2 points for significant decrease, one point for slight decrease, zero points for grossly similar, minus one point for slight increase and minus two points for significant increase, except for items 3 and 18 were grossly similar is scored as zero points, slight increase or decrease as minus one point and significant increase or decrease as minus two points. The survey was translated into Hebrew and was subjected to validation by an expert panel and pilot tested by 30 respondents who were asked to provide feedback on clarity, relevance and other aspects of the tool.

### 2.6 Statistical Analysis

Continuous data were expressed as mean ± standard deviation for normal distribution or median [IQR] for skewed distribution. Categorical variables were presented as numbers and percentages. Group differences in continuous data were assessed using independent‐sample *t*‐tests or Mann–Whitney U‐tests. Differences in categorical data were examined using Fisher's exact and Chi‐squared tests, as appropriate. The statistical analysis utilized the Kruskal‐Wallis rank sum test to evaluate differences in responses among the groups, Pearson's Chi‐squared test to scrutinize associations between categorical variables, and Fisher's exact test for smaller sample sizes. One‐Way ANOVA was utilized to check differences among genders in each profession group. Significance was determined at a *p*‐value < 0.05. The analysis was conducted using SPSS (IBM SPSS Statistics for Windows, Version 29; IBM Corp., Armonk, NY).

## 3 Results

The survey was distributed to 700 healthcare workers (HCWs) and students, resulting in 384 completed responses, with a response rate of 54.9%. The participants were divided into three groups: 65 (16.9%) were physicians, 89 (23.2%) were nurses, and 230 (59.9%) were students, of whom 93% were nursing students and 7% were medical students. Table 1 presents the demographics of each group. Among nurses and students, there was a predominance of females (*p* < 0.001), and nurses had more seniority compared to the other groups (*p* < 0.001). Families with children were more common among nurses and physicians (*p* < 0.001). Nurses and physicians tended to have more frequent night‐shift work (*p* < 0.001) and showed higher weight gain (delta weight) (*p* < 0.001) compared to students. Nurses exhibited a higher BMI, suggesting overweight, in comparison to physicians and students who reported being in the normal range at the time of completing the questionnaire. Over the course of their work or study period, nurses experienced a significant increase in BMI compared to physicians and students (*p* < 0.001). There were no significant comorbidities and no differences in the weekly physical exercise hours among the three groups. Regarding pharmacotherapy, none were undergoing weight loss treatment at the time of completing the survey, among those who did declare comorbidities, two nurses and one physician were taking aspirin as prophylaxis, one physician and one nurse were using statins, and one student was on an Angiotensin‐converting enzyme inhibitor for hypertension.

**TABLE 1:** Medical and demographic background distribution per profession.

Characteristic	Physician, *N* = 65^*a*^	Nurse, *N* = 89^*a*^	Student, *N* = 230^a^	*p* ^b^
Age (yrs)	35 (9)	36 (10)	28 (6)	**< 0.001**
Gender (female)	31/65 (48%)	74/89 (83%)	185/230 (80%)	**< 0.001**
Seniority (yrs)	8 (8)	12 (11)	2 (3)	**< 0.001**
Marital status	—	—	—	**< 0.001**
Married	38/65 (58%)	58/89 (65%)	88/230 (38%)	—
Single	26/65 (40%)	23/89 (26%)	133/230 (58%)	—
Else	1/65 (1.5%)	8/89 (9.0%)	9/230 (3.9%)	—
Children	30/65 (46%)	61/89 (69%)	78/230 (34%)	**< 0.001**
BMI	25.0 (3.8)	26.1 (4.7)	24.5 (4.7)	**0.006**
Delta BMI (now‐before)	1.61 (2.09)	2.15 (3.67)	0.46 (2.54)	**< 0.001**
Comorbidities (yes)	4/65 (6.2%)	6/89 (6.7%)	5/230 (2.2%)	0.077
Nightshift (per mo)	4.72 (2.81)	3.22 (3.51)	0.55 (2.32)	**< 0.001**
Physical exercise/week (hours)	1.8 (2.00)	1.7 (1.84)	1.94 (2.74)	0.69

*Note:* Student group includes 214 nursing students and 16 medical students.

^a^ Mean (SD); *n*/*N* (%).

^b^ Kruskal‐Wallis rank sum test; Pearson's Chi‐squared test; Fisher's exact test.

**FIGURE 1:**
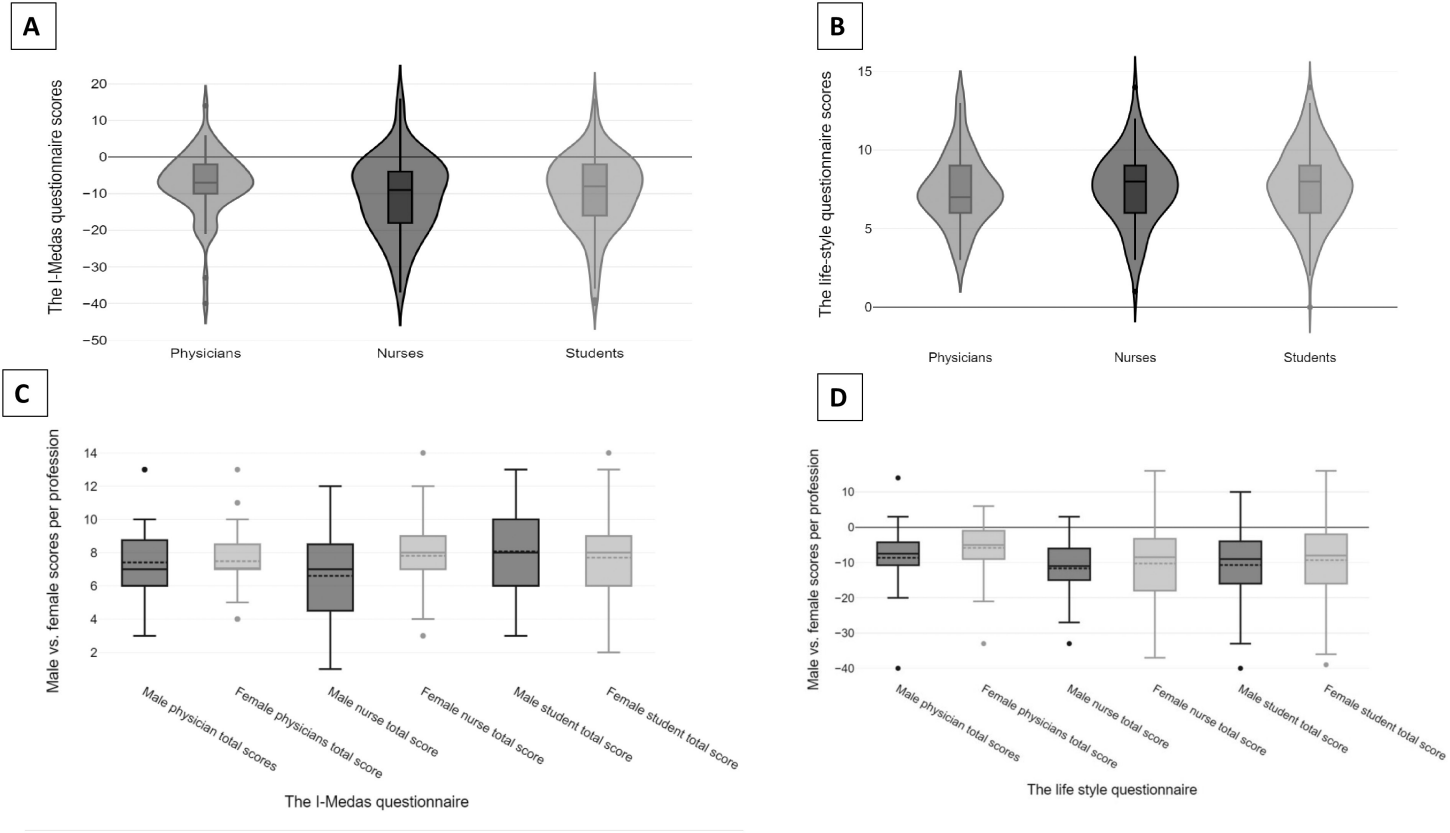
(A) The I‐MEDAS questionnaire scores among physicians, nurses and students. (B) The lifestyle related behavior questionnaire scores among physicians, nurses and students. (C) The I‐MEDAS questionnaire scores: Males versus females. (D) The lifestyle related behavior questionnaire scores: Males versus females.

Table 2 displays the I‐MEDAS questionnaire results by profession. On average, physicians scored 7 [6.0, 9.0], nurses scored 8 [6.0, 9.0], and students scored 8 [6.0, 9.0], with non‐significant *p*‐value of 0.5. Figure 1A depicts the distribution of scores from the I‐MEDAS questionnaire across various professions.

**TABLE 2:** The total scores of the questionnaires per profession.

Questionnaire	Physician, *N* = 65^a^	Nurse, *N* = 89^a^	Student, *N* = 230^a^	*p* ^b^
The I‐MEDAS questionnaire	7 [6.0, 9.0]	8 [6.0, 9.0]	8 [6.0, 9.0]	0.5
The lifestyle related behavior questionnaire	−7 [−10.0, −2.0]	−9 [−18.0, −4.0]	−8 [−16.0, −2.5]	0.11

*Note:* Student group includes 214 nursing students and 16 medical students.

^a^ Median ± IQR.

^b^ Kruskal‐Wallis rank sum test.

When analyzing specific questionnaire responses, significant differences emerged. Students reported eating three or more portions of fish per week compared to nurses and physicians (*p* = 0.022), and they consumed fewer sweet pastries per week than nurses and physicians (*p* < 0.001). Furthermore, students exhibited healthier eating pattern trends compared to nurses and physicians although no significant differences were found. They used more olive oil as their main oil, ate more whole grains, legumes, hummus salad, or tahini, and indulged less in savory pastries and salty snacks. Similar patterns were observed for unsweetened dairy products, nuts, alcohol, and red meat or processed meat between students and physicians. In addition, students and nurses tended only to consumed more butter, margarine, sweet cream, and sweetened soft drinks compared to physicians (Table 3).

**TABLE 3:** I‐MEDAS questionnaire results per profession.

Question	Physicians, *N* = 45^a^	Nurses, *N* = 67^a^	Students, *N* = 136^a^	*p* ^b^
1. Do you use olive oil as your main oil (in cooking and adding to salads/foods)?	33/45 (73%)	52/67 (78%)	109/136 (80%)	0.6
2. Do you eat poultry (chicken, turkey) more often than beef processed meat?	26/45 (58%)	46/67 (69%)	87/136 (64%)	0.5
3. How many portions of vegetables do you eat per day (1 portion = 200 g. e.g., a large tomato, a large sweet pepper, 2 medium cucumbers, 1 cup vegetable salad; addition of vegetables to a main dish = ½ portion)? 0 or 1 portion More than 2 portions	29/45 (64%) 16/45 (36%)	47/67 (70%) 20/67 (30%)	88/136 (65%) 48/136 (35%)	0.7
4. How many portions of fruit do you eat per day (Not including fruit juice) (1 portion = 125 g. e.g., medium apple, small orange, 2 tangerines, thin slice of watermelon, 3 apricots, 2 plum, small bunch of grapes, cup of cubed melon)? Up to 2 portions More than 3 portions	44/45 (98%) 1/45 (2.2%)	59/67 (88%) 8/67 (12%)	121/136 (85%) 15/136 (11%)	0.2
5. How many portions of butter, margarine, or sweet cream do you eat per day (1 portion = 12 g, or 2 teaspoons; light layer on one slice of bread is ½ a portion)? 0 or 1 portion More than 2 portions	43/45 (96%) 2/45 (4.4%)	56/67 (88%) 11/67 (16%)	116/136 (85%) 20/136 (15%)	0.15
6. How many cups of sweetened soft drinks (e.g., fruit‐flavored soft drinks, energy drinks, regular [not diet] cola/carbonated drinks) do you drink per day? 0 or 1 portion More than 2 portions	38/45 (84%) 7/45 (16%)	50/67 (75%) 17/67 (25%)	98/136 (72%) 38/136 (28%)	0.2
7. How many portions of whole grains do you eat per day? (e.g., whole‐grain bread or pasta, burgul, ferikeh, buckwheat, brown rice, oats) (1 portion = 1 slice of bread or ½ cup cooked grains) Up to 2 portions More than 3 portions	40/45 (89%) 5/45 (11%)	60/67 (90%) 7/67 (10%)	111/136 (82%) 25/136 (18%)	0.2
8. How many portions of unsweetened dairy products do you eat per day? (e.g., milk, cheese, yogurt, labaneh, white/cottage cheese) (1 portion = ½ cup of milk, small container of yogurt, 75 g salty white cheese, processed cheese triangle…) 0 or 1 portion More than 2 portions	30/45 (67%) 15/45 (33%)	35/67 (52%) 32/67 (48%)	91/136 (67%) 45/136 (33%)	0.11
9. How many portions of red meat (e.g., beef or lamb grilled meat/steak) hamburger or processed meat (e.g., salami, hot dogs) do you eat per week? (1 portion = 100‐150 g red meat or 60 g processed meat; e.g., 1 hamburger or 2 hot dogs) Up to 6 meals More than 7 meals	41/45 (91%) 4/45 (9%)	60/67 (90%) 7/67 (10%)	123/136 (90%) 13/136 (10%)	> 0.9
10. How many drinks of alcohol do you have per week? (1 portion = glass of wine, glass or can of beer, shot glass of hard liquor like whiskey, vodka) Up to 6 meals More than 7 meals	43/45 (96%) 2/45 (4.4%)	61/67 (91%) 6/67 (9.0%)	130/136 (96%) 6/136 (4.4%)	0.4
11. How many portions of legumes (e.g., lentils, white beans, garbanza beans, turmos, fava beans) do you eat per week? (1 portion = 150 g or ¾ cup of cooked legumes) Up to 2 portions More than 3 portions	35/45 (78%) 10/45 (22%)	55/67 (82%) 12/67 (18%)	103/136 (76%) 33/136 (24%)	0.6
12. How many portions of fish do you eat per week? (1 portion = 100–150 g fresh fish, or 1 can of tuna, or 60 g smoked/pickled fish, or 10 shrimps) Up to 2 portions More than 3 portions	43/45 (96%) 2/45 (4.4%)	59/67 (88%) 8/67 (12%)	108/136 (79%) 28/136 (21%)	0.022
13. How many portions of nuts do you eat per week? (1 portion = handful, 30 g) Up to 2 portions More than 3 portions	34/45 (76%) 11/45 (24%)	49/67 (73%) 18/67 (27)	109/136 (80%) 27/136 (20%)	0.5
14. How many portions of hummus salad or tahini do you eat per week? (including tahini added to cooked foods, and hummus and tahini salads) (1 portion = 1 tablespoon) Up to 2 portions More than 3 portions	32/45 (76%) 13/45 (29%)	48/67 (72%) 19/67 (28%)	91/136 (67%) 45/136 (33%)	0.7
15. How many times per week do you eat sweet pastries? (store bought or homemade), (e.g., cakes, wafers, biscuits/cookies, kanafa, baklawa). Up to 2 portions More than 3 portions	17/45 (38%) 28/45 (62%)	26/67 (39%) 41/67 (61%)	87/136 (67%) 45/136 (33%)	< 0.001
16. How many portions of savory pastries do you eat per week? (e.g., burekas, jahnoun, malaweh) (1 portion = 50–60 g or 1 burekas) Up to 2 portions More than 3 portions	32/45 (71%) 13/45 (29%)	47/67 (70%) 20/67 (30%)	107/136 (79%) 29/136 (21%)	0.3
17. How many portions of salty snacks do you eat per week? (e.g., peanut buttercoated corn puffs, potato chips) (1 portion = 25 g, 1 small snack bag) Up to 2 portions More than 3 portions	29/45 (64%) 16/45 (36%)	49/67 (73%) 18/67 (27%)	107/136 (79%) 29/136 (21%)	0.2

^a^ *n*/*N* (%).

^b^ Pearson's Chi‐squared test; Fisher's exact test.

When assessing the results of the lifestyle related behavior questionnaire response, no significant differences were found between physicians, nurses and students (*p* = 0.11) although the group of nurses received lower scores (Table 2). Figure 1B depicts the distribution of scores from the lifestyle questionnaire across various professions.

Table 4, illustrates the percentage of participants who provided relevant answers per profession. The data indicated changes in lifestyle among all groups from the start of the studies period or the beginning of the period of work profession. In all professions (physicians vs. nurses vs. students) an increase was reported in the probability of skipping main meals (87.69% vs. 85.39% vs. 71.11%, respectively), more snacking between meals (75.38% vs. 69.66% vs. 60.44%, respectively), increased quantities of meals and snacks (69.23% vs. 71.59% vs. 58.26%, respectively), processed food consumption (73.85% vs. 56.18% vs. 49.31%, respectively), sugar‐sweetened juice intake (57.81% vs. 45.98% vs. 45.58%, respectively), candy and chocolate consumption (72.31% vs. 64.77% vs. 51.46%, respectively), more sedentary behavior and screen time (56.92% vs. 49.41% vs. 53.78%, respectively), and elevated levels of stress and anxiety (68.75% vs. 70.93% vs. 67.56%, respectively). Furthermore, it's worth noting that a decrease was reported in the consumption of fruits and vegetables (45.31% vs. 31.82% vs. 21.17%, respectively), as well as a reduced adherence to a balanced diet comprising elements like whole wheat, pulses, legumes, eggs, nuts, fruits, and vegetables (44.62% vs. 31.03% vs. 21.52%, respectively). Additionally, physicians and nurses reported lower levels of participation in aerobic exercise when compared to students (30.77% vs. 32.66% vs. 23.11%, respectively), leisure activities and household chores (50.77% vs. 31.40% vs. 27.60%, respectively), additionally they reported fewer hours of sleep (52.31% vs. 42.53% vs. 41.59%, respectively) and lower sleep quality (53.85% vs. 46.51% vs. 36.65%, respectively), Table 4.

**TABLE 4:** Percentage distribution of respondents to the lifestyle questionnaire based on professions.

	Decreased			Increased		
Questions	Physicians	Nurses	Students	Physicians	Nurses	Students
1. How has your probability of skipping one of the main meals (breakfast/lunch/dinner) changed?	4.62	3.37	15.56	87.69	85.39	71.11
2. How has your habit of snacking between meals changed?	7.69	10.11	19.56	75.38	69.66	60.44
3. How has your quantity/portions of meals and snacks changed?	10.77	14.77	18.26	69.23	71.59	58.26
4. How has your daily intake of fruits and vegetables changed?	45.31	31.82	21.17	21.88	37.50	41.44
5. How has your intake of a balanced diet (including healthy ingredients such as whole wheat, pulses, legumes, eggs, nuts, fruits and vegetables) changed?	44.62	31.03	21.52	26.15	41.38	38.57
6. How has your consumption of junk food/fast food and fried food changed?	7.69	19.10	15.67	73.85	56.18	49.31
7. How your intake of sugar‐sweetened beverages (carbonated soft drinks, sugar‐sweetened juices) changed?	15.63	25.29	19.07	57.81	45.98	45.58
8. How has your consumption of sweets/candies/chocolate changed?	9.23	13.64	17.48	72.31	64.77	51.46
9. How has your participation in cooking new/traditional recipes changed?	72.31	50.00	39.13	9.23	23.81	20.77
10. How has your consumption of unhealthy food when you are bored or stressed or upset changed?	46.03	18.39	12.67	15.87	60.92	59.28
11. How has your intake of immunity‐boosting foods (lemon, turmeric, garlic, citrus fruits and green leafy vegetables) in the diet changed?	4.62	20.45	13.39	64.62	40.91	33.04
12. How has your intake of nutrition supplements to boost immunity changed?	20.00	16.05	12.16	16.92	29.63	30.63
13. How has the support of your family and friends in eating healthy changed?	15.38	11.63	10.22	6.15	39.53	41.78
14. How has your interest in learning healthy eating tips from the media (newspaper articles/magazines blogs/videos/TV shows/text messages) changed?	21.54	23.86	12.67	23.08	45.45	49.77
15. How has your participation in aerobic exercise changed?	30.77	32.56	23.11	26.15	32.56	30.66
16. How has your participation in leisure and household chores changed?	50.77	31.40	27.60	15.38	45.35	36.20
17. How has your sitting and screen time changed?	26.15	22.35	22.22	56.92	49.41	53.78
18. How have your hours of sleep changed?	52.31	42.53	41.59	15.38	40.23	36.73
19. How has your quality of sleep changed?	53.85	46.51	36.65	20.00	37.21	38.46
20. How have your stress and anxiety levels changed?	9.38	9.30	8.89	68.75	70.93	67.56

Figure 1C showcases the box plot of I‐MEDAS scores among different professions, further categorized by gender within each profession group. No significant differences were found between genders male versus female in all groups (*p* = 0.25) yet higher scores were obtained by female nurses, as well as male and female students.

Figure 1D illustrates the box plot of lifestyle related behavior questionnaire scores across various professions, with classification by gender within each profession group. No significant differences were found between genders male versus female in all groups (*p* = 0.35), although females in each group showed better scores especially among physicians.

The regression analysis unveiled significant correlations, indicating that unmarried physicians exhibited better scores in the lifestyle related behavior questionnaire (*p* = 0.003), particularly those without children (*p* = 0.002). Furthermore, marital status emerged as a correlating factor for I‐MEDAS scores, with married physicians showing a significant association (*p* = 0.008).

## 4 Discussion

Health outcomes are significantly influenced by nutrition and lifestyle choices. Despite this, there is a limited amount of research examining these behaviors in nursing and medical students as compared to HCWs. In our study, as anticipated, nurses and physicians demonstrated more frequent night‐shift work and higher weight gain compared to students. Nurses experienced significant increase in BMI compared to both physicians and students. While there were no significant overall differences in total scores between the groups in the two questionnaires, a median score of 7 or 8 (out of 17) in the I‐MEDAS questionnaire implies suboptimal adherence to the Mediterranean diet in a Mediterranean country. This is noteworthy as it aligns with the general population scores in Israel (Abu‐Saad et al. 2019), emphasizing that knowledge about a healthy diet might not be a decisive factor in dietary choices. Moreover, a median score of −7, −8, or −9 in the lifestyle related behavior questionnaire indicates a shift in lifestyle behaviors toward more negative health behaviors after the initiation of work or studies, this might be attributed to high workload, shift work, limited access to healthy meal options, emotional exhaustion, and the imbalance between work and personal life. Adherence to the Mediterranean diet remained unaffected by gender; however, female nurses and both male and female students achieved higher scores. Conversely, females exhibited a trend of better scores in the lifestyle related behavior questionnaire, especially among physicians.

There's a lack of data assessing lifestyle changes during the transitional period from college to work. An Israeli study (Wilf‐Miron, Kagan, and Saban 2021) which revealed that medical students tend to adopt a healthy lifestyle at the onset of medical school, showed that this behavior gradually declines over time, becoming less healthy during their residency. In contrast, our study did not uncover notable variations in questionnaire scores between students and HCWs, which may indicate a consistent pattern of negative food choices and lifestyle habits from the commencement of their studies. Surprisingly, there was an absence of the expected shift toward healthier practices over time, despite the accumulation of knowledge regarding the benefits of nutritious diets and improved lifestyle habits. This shift is warranted, especially in the post‐COVID‐19 era that has prompted various conclusions, particularly concerning the support and well‐being of HCWs (Shreffler, Petrey, and Huecker 2020). Acknowledging the importance of preventive measures, there has been an increased emphasis on strategies to encourage HCWs to prioritize their health (Søvold et al. 2021).

Moreover, there are several gaps in the literature regarding the long‐term influence of healthcare education on nurses' and physicians' self‐health behaviors. Most studies focus on short‐term impacts, with limited longitudinal data on how these behaviors evolve throughout their careers (Ross et al. 2017, 2019; Wills, Hancock, and Nuttall 2020). Research on the effectiveness of specific educational interventions, such as courses on stress management and nutrition, are also lacking. Additionally, there is minimal comparison between how nurses and physicians adopt and maintain healthy behaviors over time (Fie, Norman, and While 2012), as well as insufficient exploration of cultural and regional differences in healthcare education's impact. Furthermore, the role of workplace factors, such as shift work and institutional support, in sustaining healthy behaviors learned during training remains underexplored. Future studies are warranted in addressing these gaps to enhance the understanding and the improvements in educational and workplace interventions.

The nursing community faces significant workplace hazards, including poor work ergonomics, staff shortages, inadequate team leadership, and equipment insufficiencies, contributing to heightened stress levels and increased risks of occupational diseases (Feo, Kitson, and Conroy 2018). These challenges, coupled with limited free time and fatigue, result in negative health behaviors such as the consumption of unhealthy foods, and discouraging physical activity (Babapour, Gahassab‐Mozaffari, and Fathnezhad‐Kazemi 2022). (Tucker et al. 2010) found that only 50% of hospital‐based RNs adhered to Centres for Disease Control and Prevention guidelines for physical activity, and 62% consumed fast food at least twice weekly. In another survey of British nurses, less than half met government guidelines for physical activity, and over half reported daily consumption of high‐fat, sugary foods (Blake et al. 2011). A recent study involving Israeli nurses revealed that two‐thirds did not meet physical activity targets, especially those working night shifts (Kagan et al. 2022). The prevalence of overweight nurses ranges from 30% to 53%, comparable to or higher than the general population (Buss 2012). Over half of nurses in Australia, New Zealand, and the UK were found to be overweight or obese, surpassing rates in the general population (Bogossian et al. 2012). All participants in the current survey had a BMI status in the upper normal range, and notably, nurses specifically registered a BMI score in the overweight range, in addition to the lack of proper exercise regimen of 150 minutes weekly (American Heart Association n.d.) which increase the risk for further weight gain, obesity, and other related conditions.

Numerous challenges impact food and eating habits among nurses, including unhealthy workplace social eating practices, issues of food availability, and variable food quality (with vending machines predominantly supplying junk food and cafés offering menus of questionable quality in facilities not conducive to staff comfort) (Nicholls et al. 2017). In Australia (Perry et al. 2018), a cross‐sectional survey involving 5041 nurses revealed concerning lifestyle behaviors. Older nurses tended to follow fruit and vegetable guidelines more, but they were also more likely to be overweight. Many nurses displayed negative health behaviors that put them at high risk for non‐communicable diseases, sometimes exceeding the risk observed in the general Australian population. In our study, there is an observed increase in skipping main meals, more snacking between meals, higher quantities of meals and snacks, increased processed food consumption, elevated sugar‐sweetened juice intake, and higher levels of candy and chocolate consumption across all professions. This could be attributed to high workloads, irregular and long working or studying hours, and the demands of after studies work or shift work. These factors disrupt normal eating patterns and leave little time for proper meals. Stress, time constraints, and a fast‐paced environment also contribute to a reliance on snacks, which are often more convenient. In our study, physicians may consume more junk food and sugar‐sweetened beverages compared to nurses and students possibly due to longer working hours, higher stress levels, and greater work responsibility, which often lead to convenience‐based food choices.

To encourage regular, healthy meals, workplace policies could be adapted to include structured meal breaks that are protected from work interruptions. Healthcare facilities can provide healthy food options on‐site, and ensure that food is available during night shifts. Flexible scheduling that accommodates proper meal times, as well as initiatives promoting mindfulness around self‐care, could also help reduce the frequency of meal skipping and improve overall well‐being for healthcare workers. Dietary interventions can lead to meaningful weight loss both in the short and long term, potentially benefiting nurses, among whom obesity and central adiposity are prevalent (Greaves et al. 2011). However, interventions specifically tailored for the nursing workforce are scarce (Chan and Perry 2012), highlighting a potential area for future interventions, particularly targeting younger nurses.

Maintaining health and well‐being relies significantly on adequate sleep. Insufficient sleep duration and poor sleep quality which were reported by nurses and physicians in our study are linked to various adverse health outcomes, including an elevated risk of weight gain, obesity, hypertension, diabetes, cardiovascular disease, and depression (Watson et al. 2015). The reported decrease in sleep quality and hours is closely linked to their elevated stress and anxiety levels. Irregular shifts, long working hours, and emotional demands of patient care disrupt natural sleep patterns, leading to chronic sleep deprivation. This lack of sleep exacerbates stress and anxiety, creating a cycle of poor mental and physical health (Gerace and Rigney 2020; Okechukwu et al. 2023). To improve sleep hygiene, workplace changes such as implementing more consistent shift schedules, providing rest breaks, offering stress management resources, and promoting a supportive environment for mental health could help healthcare workers achieve better sleep and overall well‐being (Zhang et al. 2023). Effective stress management resources for healthcare workers include mindfulness‐based programs, cognitive behavioral therapy workshops, and peer support groups (Chesak et al. 2019). In addition, integrating relaxation techniques like meditation and yoga can also promote better emotional well‐being and sleep hygiene. Collectively, unhealthy nutrition and lifestyle behaviors, including decreased sleep hours and quality, emerge as potential environmental contributors to the spectrum of metabolic syndrome components in HCWs.

HCWs serve as a dependable, confidential, and knowledgeable source of advice across various health‐related subjects. Their unique position allows them to play a pivotal role in promoting healthy lifestyle habits among their patients. Physicians who maintain healthy habits are more likely to engage in discussions about them with their patients, facilitating effective dialogue and motivating patients to adopt healthier lifestyles (Frank et al. 2010; Howe et al. 2010). The credibility of physicians' advice is enhanced when they disclose their own health behaviors to patients (Frank, Breyan, and Elon 2000).

To our knowledge, this is the first cohort study that compared the health behavior of nursing and medical students and compares it to HCWs. Establishing a work environment and culture that prioritizes health and well‐being across the entire spectrum of professional training, spanning from nursing and medical school through residency to senior physician/ nursing roles, holds substantial advantages. This proactive approach extends benefits all health professionals sharing the same work milieu, patients, their families, and the broader healthcare system. By fostering a culture that values and supports well‐being at every career stage, we can enhance not just individual practitioner health but also the overall quality of patient care and the efficiency of healthcare delivery.

Concerning limitations, our study acknowledges the small sample size and the potential for volunteer bias as our sample was drawn from volunteers, introducing the possibility that participants were inherently health‐conscious and motivated. This convenience sampling method could have attracted individuals actively seeking opportunities to enhance their well‐being, possibly skewing the results. It's plausible that those who volunteered were more aware of their health needs and, thus, more inclined to participate. The recruitment method of distributing the survey via email or phone may introduce nonresponse bias, as those with greater access to technology or less demanding schedules may be more likely to respond. However, the study attempted to minimize this bias by allowing participants flexibility in how they could complete the survey, providing both email and phone message options. Additionally, participation was voluntary, and while completing the entire survey was encouraged, it was not mandatory, allowing for partial responses to still be included in the analysis. This approach helped accommodate different levels of availability and technology access, potentially broadening the range of respondents and reducing the impact of nonresponse bias. It is essential to note, that the data in this study are observational, and inherent weaknesses include those associated with self‐reported dietary and physical exercise information. Despite controlling for confounding variables such as demographic, socioeconomic factors, health status, and lifestyle parameters in the analyses, the potential for confounding by unmeasured or untested factors cannot be entirely ruled out. In addition, the sample size constraints limited our capacity to conduct subgroup analyses, both in terms of population groups and specific food components, especially in cases where intake was minimal. Additionally, since our study focused on Israel, generalizing these findings should be approached with caution. Replication and extension studies in diverse geographic regions are essential to validate and broaden the applicability of our observations. This approach ensures a more comprehensive understanding of health behaviors that transcends specific cultural contexts.

## 5 Conclusions

Overall questionnaire scores highlighted a negative change in the lifestyle behaviors and suboptimal adherence to the Mediterranean diet among HCWs and students, with no gender differences. Moreover, nurses and physicians stood out with more frequent night‐shift work and higher weight gain than students. Notably, nurses experienced a significant increase in BMI and reported reduced engagement in leisure activities, household chores, fewer hours of sleep, and lower sleep quality. To sustain their health amid the demanding nature of their work, HCWs should receive ongoing support and resources. This not only shields them from job‐related health risks but also enables them to set an example for patients. Such initiatives contribute to the well‐being of both healthcare professionals and the entire healthcare system.

## 6 Relevance for Clinical Practice

Based on the findings of this study, understanding the distinct lifestyle practices and dietary preferences of nurses, physicians, and nursing students can help inform targeted interventions to address their unique health challenges. Tailored support initiatives, such as workplace wellness programs and stress management resources, can enhance the overall health and well‐being of healthcare professionals, especially nursing students who may face additional stress during training. Implementing nutritional education early in nursing programs may further encourage healthier habits. These strategies are vital for improving both personal well‐being and the quality of care provided by healthcare workers.

## Author Contributions

**Hussein Zaitoon:** conceptualization, investigation, writing – original draft, methodology, writing – review and editing, visualization, project administration, data curation, resources, software. **Lisa Kaly:** validation, software, formal analysis, data curation. **Hadel Khalil:** conceptualization, investigation, methodology, data curation, project administration, writing – review and editing. **Nataly Zion:** project administration, supervision, methodology, conceptualization, investigation, writing – review and editing, resources, validation.

## Conflicts of Interest

The authors declare no conflicts of interest.

## Ethics Statement

The study was approved by the Ethics Committee of the Bnai Zion Medical Center according to the Helsinki Declaration (approval number 0054‐22‐BNZ). This study was performed in line with the principles of the Declaration of Helsinki.

## Consent

Participants were informed about the study and asked to provide their consent before filling out the questionnaire.

## Supporting information


Data S1.

## Data Availability

The data that support the findings of this study are available from the corresponding author upon reasonable request.

## References

[nhs70000-bib-0001] Abu‐Saad, K. , R. Endevelt , R. Goldsmith , et al. 2019. “Adaptation and Predictive Utility of a Mediterranean Diet Screener Score.” Clinical Nutrition 38, no. 6: 2928–2935. 10.1016/J.CLNU.2018.12.034. 30642736

[nhs70000-bib-0002] Amani, R. , and T. Gill . 2013. “Shiftworking, Nutrition and Obesity: Implications for Workforce Health‐ a Systematic Review.” Asia Pacific Journal of Clinical Nutrition 22, no. 4: 505–515. 10.6133/APJCN.2013.22.4.11. 24231009

[nhs70000-bib-0003] American Heart Association . n.d. “American Heart Association Recommendations for Physical Activity in Adults and Kids.” Retrieved November 28, 2023. https://www.heart.org/en/healthy‐living/fitness/fitness‐basics/aha‐recs‐for‐physical‐activity‐in‐adults.

[nhs70000-bib-0004] Babapour, A. R. , N. Gahassab‐Mozaffari , and A. Fathnezhad‐Kazemi . 2022. “Nurses' Job Stress and Its Impact on Quality of Life and Caring Behaviors: A Cross‐Sectional Study.” BMC Nursing 21, no. 1: 1–10. 10.1186/S12912-022-00852-Y/TABLES/5. 35361204 PMC8968092

[nhs70000-bib-0005] Blake, H. , S. Malik , P. K. H. Mo , and C. Pisano . 2011. ““Do as Say, but Not as I Do”: Are Next Generation Nurses Role Models for Health?” Perspectives in Public Health 131, no. 5: 231–239. 10.1177/1757913911402547. 21999028

[nhs70000-bib-0006] Bogossian, F. E. , J. Hepworth , G. M. Leong , et al. 2012. “A Cross‐Sectional Analysis of Patterns of Obesity in a Cohort of Working Nurses and Midwives in Australia, New Zealand, and the United Kingdom.” International Journal of Nursing Studies 49, no. 6: 727–738. 10.1016/J.IJNURSTU.2012.01.003. 22307023

[nhs70000-bib-0007] Buchvold, H. V. , S. Pallesen , N. M. F. Øyane , and B. Bjorvatn . 2015. “Associations Between Night Work and BMI, Alcohol, Smoking, Caffeine and Exercise–a Cross‐Sectional Study.” BMC Public Health 15, no. 1: 1112. 10.1186/S12889-015-2470-2. 26558686 PMC4642677

[nhs70000-bib-0008] Buss, J. 2012. “Associations Between Obesity and Stress and Shift Work Among Nurses.” Workplace Health & Safety 60, no. 10: 453–458. 10.1177/216507991206001007. 23054165

[nhs70000-bib-0009] Cena, H. , and P. C. Calder . 2020. “Defining a Healthy Diet: Evidence for the Role of Contemporary Dietary Patterns in Health and Disease.” Nutrients 12, no. 2: 334. 10.3390/NU12020334. 32012681 PMC7071223

[nhs70000-bib-0010] Chan, C. W. , and L. Perry . 2012. “Lifestyle Health Promotion Interventions for the Nursing Workforce: A Systematic Review.” Journal of Clinical Nursing 21, no. 15–16: 2247–2261. 10.1111/J.1365-2702.2012.04213.X. 22788559

[nhs70000-bib-0011] Chesak, S. S. , S. M. Cutshall , C. L. Bowe , K. M. Montanari , and A. Bhagra . 2019. “Stress Management Interventions for Nurses: Critical Literature Review.” Journal of Holistic Nursing 37, no. 3: 288–295. 10.1177/0898010119842693/ASSET/IMAGES/LARGE/10.1177_0898010119842693-FIG1.JPEG. 31014156

[nhs70000-bib-0012] Dominguez, L. J. , G. Di Bella , N. Veronese , and M. Barbagallo . 2021. “Impact of Mediterranean Diet on Chronic Non‐Communicable Diseases and Longevity.” Nutrients 13, no. 6: 2028. 10.3390/NU13062028. 34204683 PMC8231595

[nhs70000-bib-0013] Emilien, C. , and J. H. Hollis . 2017. “A Brief Review of Salient Factors Influencing Adult Eating Behaviour.” Nutrition Research Reviews 30, no. 2: 233–246. 10.1017/S0954422417000099. 28625227

[nhs70000-bib-0014] Feo, R. , A. Kitson , and T. Conroy . 2018. “How Fundamental Aspects of Nursing Care Are Defined in the Literature: A Scoping Review.” Journal of Clinical Nursing 27, no. 11–12: 2189–2229. 10.1111/JOCN.14313. 29514402

[nhs70000-bib-0015] Fie, S. , I. J. Norman , and A. E. While . 2012. “The Relationship Between physicians' and nurses' Personal Physical Activity Habits and Their Health‐Promotion Practice: A Systematic Review.” Health Education Journal 72, no. 1: 102–119. 10.1177/0017896911430763.

[nhs70000-bib-0016] Frank, E. , J. Breyan , and L. Elon . 2000. “Physician Disclosure of Healthy Personal Behaviors Improves Credibility and Ability to Motivate.” Archives of Family Medicine 9, no. 3: 287–290. 10.1001/ARCHFAMI.9.3.287. 10728118

[nhs70000-bib-0017] Frank, E. , C. Segura , H. Shen , and E. Oberg . 2010. “Predictors of Canadian physicians' Prevention Counseling Practices.” Canadian Journal of Public Health 101, no. 5: 390–395. 10.1007/BF03404859. 21214054 PMC6974278

[nhs70000-bib-0018] Gao, Y. , T. Gan , L. Jiang , et al. 2020. “Association Between Shift Work and Risk of Type 2 Diabetes Mellitus: A Systematic Review and Dose‐Response Meta‐Analysis of Observational Studies.” Chronobiology International 37, no. 1: 29–46. 10.1080/07420528.2019.1683570. 31684766

[nhs70000-bib-0019] Gerace, A. , and G. Rigney . 2020. “Considering the Relationship Between Sleep and Empathy and Compassion in Mental Health Nurses: It's Time.” International Journal of Mental Health Nursing 29, no. 5: 1002–1010. 10.1111/INM.12734. 32406147

[nhs70000-bib-0020] Greaves, C. J. , K. E. Sheppard , C. Abraham , et al. 2011. “Systematic Review of Reviews of Intervention Components Associated With Increased Effectiveness in Dietary and Physical Activity Interventions.” BMC Public Health 11, no. 1: 1–12. 10.1186/1471-2458-11-119/TABLES/2. 21333011 PMC3048531

[nhs70000-bib-0021] Howe, M. , A. Leidel , S. M. Krishnan , A. Weber , M. Rubenfire , and E. A. Jackson . 2010. “Patient‐Related Diet and Exercise Counseling: Do providers' Own Lifestyle Habits Matter?” Preventive Cardiology 13, no. 4: 180–185. 10.1111/J.1751-7141.2010.00079.X. 20860642

[nhs70000-bib-0022] Kagan, I. , A. Ziv , C. Rubin , et al. 2022. “Effect of Ethnicity, Country of Origin and Workplace on Health Behaviors and Health Perception Among Nurses: Cross‐Sectional Study.” Journal of Nursing Scholarship 54, no. 5: 535–545. 10.1111/JNU.12759. 34951740

[nhs70000-bib-0023] Kritsotakis, G. , E. D. Georgiou , G. Karakonstandakis , N. Kaparounakis , V. Pitsouni , and P. Sarafis . 2020. “A Longitudinal Study of Multiple Lifestyle Health Risk Behaviours Among Nursing Students and Non‐nursing Peers.” International Journal of Nursing Practice 26, no. 6: e12852. 10.1111/IJN.12852. 32645751

[nhs70000-bib-0024] Kumari, A. , P. Ranjan , N. K. Vikram , et al. 2020. “A Short Questionnaire to Assess Changes in Lifestyle‐Related Behaviour During COVID 19 Pandemic.” Diabetes and Metabolic Syndrome: Clinical Research and Reviews 14, no. 6: 1697–1701. 10.1016/J.DSX.2020.08.020. PMC7448879 32911201

[nhs70000-bib-0025] Lowden, A. , C. Moreno , U. Holmbäck , M. Lennernäs , and P. Tucker . 2010. “Eating and Shift Work ‐ Effects on Habits, Metabolism and Performance.” Scandinavian Journal of Work, Environment & Health 36, no. 2: 150–162. 10.5271/SJWEH.2898. 20143038

[nhs70000-bib-0026] Macedo, T. T. S. , F. C. Mussi , D. Sheets , et al. 2020. “Lifestyle Behaviors Among Undergraduate Nursing Students: A Latent Class Analysis.” Research in Nursing & Health 43, no. 5: 520–528. 10.1002/NUR.22064. 32797687

[nhs70000-bib-0027] Ministry of Health . n.d. “Mediterranean Diet.” Retrieved November 28, 2023. https://www.health.gov.il/English/Topics/FoodAndNutrition/Nutrition/Adequate_nutrition/mediterranean/Pages/default.aspx.

[nhs70000-bib-0028] Mkanzi, N. , D. T. Goon , U. B. Okafor , and E. O. Owolabi . 2020. “Screening for Cardio‐Metabolic Risk Factors Among Student Nurses: A Cross‐Sectional Study.” Global Journal of Health Science 12, no. 2: p1. 10.5539/GJHS.V12N2P1.

[nhs70000-bib-0029] Nicholls, R. , L. Perry , C. Duffield , R. Gallagher , and H. Pierce . 2017. “Barriers and Facilitators to Healthy Eating for Nurses in the Workplace: An Integrative Review.” Journal of Advanced Nursing 73, no. 5: 1051–1065. 10.1111/JAN.13185. 27732741

[nhs70000-bib-0030] Okechukwu, C. E. , C. Colaprico , S. Di Mario , et al. 2023. “The Relationship Between Working Night Shifts and Depression Among Nurses: A Systematic Review and Meta‐Analysis.” Health 11, no. 7: 937. 10.3390/HEALTHCARE11070937. PMC10094007 37046864

[nhs70000-bib-0031] Pallesen, S. , B. Bjorvatn , S. Waage , A. Harris , and D. Sagoe . 2021. “Prevalence of Shift Work Disorder: A Systematic Review and Meta‐Analysis.” Frontiers in Psychology 12: 638252. 10.3389/FPSYG.2021.638252/BIBTEX. 33833721 PMC8021760

[nhs70000-bib-0032] Perry, L. , X. Xu , R. Gallagher , R. Nicholls , D. Sibbritt , and C. Duffield . 2018. “Lifestyle Health Behaviors of Nurses and Midwives: The ‘Fit for the Future’ Study.” International Journal of Environmental Research and Public Health 15, no. 5: 945. 10.3390/IJERPH15050945. 29747412 PMC5981984

[nhs70000-bib-0033] Romero‐Blanco, C. , A. Hernández‐Martínez , M. L. Parra‐Fernández , M. D. Onieva‐Zafra , M. D. C. Prado‐Laguna , and J. Rodríguez‐Almagro . 2022. “Food Preferences in Undergraduate Nursing Students and Its Relationship With Food Addiction and Physical Activity.” International Journal of Environmental Research and Public Health 19, no. 7: 3858. 10.3390/IJERPH19073858. 35409543 PMC8998007

[nhs70000-bib-0034] Ross, A. , M. Bevans , A. T. Brooks , S. Gibbons , and G. R. Wallen . 2017. “Nurses and Health‐Promoting Behaviors: Knowledge May Not Translate Into Self‐Care.” AORN Journal 105, no. 3: 267–275. 10.1016/J.AORN.2016.12.018. 28241948 PMC5536335

[nhs70000-bib-0035] Ross, A. , L. Yang , L. Wehrlen , A. Perez , N. Farmer , and M. Bevans . 2019. “Nurses and Health‐Promoting Self‐Care: Do We Practice What We Preach?” Journal of Nursing Management 27, no. 3: 599–608. 10.1111/JONM.12718. 30223297 PMC6421110

[nhs70000-bib-0036] Shreffler, J. , J. Petrey , and M. Huecker . 2020. “The Impact of COVID‐19 on Healthcare Worker Wellness: A Scoping Review.” Western Journal of Emergency Medicine 21, no. 5: 1059–1066. 10.5811/WESTJEM.2020.7.48684. 32970555 PMC7514392

[nhs70000-bib-0037] Søvold, L. E. , J. A. Naslund , A. A. Kousoulis , et al. 2021. “Prioritizing the Mental Health and Well‐Being of Healthcare Workers: An Urgent Global Public Health Priority.” Frontiers in Public Health 9: 679397. 10.3389/FPUBH.2021.679397/BIBTEX. 34026720 PMC8137852

[nhs70000-bib-0038] Thwaite, T. L. , P. Heidke , S. L. Williams , C. Vandelanotte , A. L. Rebar , and S. Khalesi . 2020. “Barriers to Healthy Lifestyle Behaviors in Australian Nursing Students: A Qualitative Study.” Nursing & Health Sciences 22, no. 4: 921–928. 10.1111/NHS.12749. 32533602

[nhs70000-bib-0039] Torquati, L. , G. I. Mielke , W. J. Brown , and T. Kolbe‐Alexander . 2018. “Shift Work and the Risk of Cardiovascular Disease. A Systematic Review and Meta‐Analysis Including Dose–Response Relationship.” Scandinavian Journal of Work, Environment & Health 44, no. 3: 229–238. 10.5271/sjweh.3700. 29247501

[nhs70000-bib-0040] Tucker, S. J. , M. R. Harris , T. B. Pipe , and S. R. Stevens . 2010. “Nurses' Ratings of Their Health and Professional Work Environments.” AAOHN Journal: Official Journal of the American Association of Occupational Health Nurses 58, no. 6: 253–267. 10.3928/08910162-20100526-03. 20677722

[nhs70000-bib-0041] Tuttolomondo, A. , I. Simonetta , M. Daidone , A. Mogavero , A. Ortello , and A. Pinto . 2019. “Metabolic and Vascular Effect of the Mediterranean Diet.” International Journal of Molecular Sciences 20, no. 19: 4716. 10.3390/IJMS20194716. 31547615 PMC6801699

[nhs70000-bib-0042] Watson, N. F. , M. S. Badr , G. Belenky , et al. 2015. “Recommended Amount of Sleep for a Healthy Adult: A Joint Consensus Statement of the American Academy of Sleep Medicine and Sleep Research Society.” Sleep 38, no. 6: 843–844. 10.5665/SLEEP.4716. 26039963 PMC4434546

[nhs70000-bib-0043] Wilf‐Miron, R. , I. Kagan , and M. Saban . 2021. “Health Behaviors of Medical Students Decline Towards Residency: How Could We Maintain and Enhance These Behaviors Throughout Their Training.” Israel Journal of Health Policy Research 10, no. 1: 1–8. 10.1186/S13584-021-00447-Z/TABLES/2. 33866965 PMC8054363

[nhs70000-bib-0044] Wills, J. , C. Hancock , and M. Nuttall . 2020. “The Health of the Nursing Workforce. A Survey of National Nurse Associations.” International Nursing Review 67, no. 2: 294–299. 10.1111/INR.12586. 32367661

[nhs70000-bib-0045] World Health Organization . n.d. “Fostering healthier and more sustainable diets – learning from the Mediterranean and New Nordic experience.” Retrieved November 28, 2023. https://www.who.int/europe/news/item/07‐05‐2018‐fostering‐healthier‐and‐more‐sustainable‐diets‐learning‐from‐the‐mediterranean‐and‐new‐nordic‐experience.

[nhs70000-bib-0046] Zazpe, I. , R. Estruch , E. Toledo , et al. 2010. “Predictors of Adherence to a Mediterranean‐Type Diet in the PREDIMED Trial.” European Journal of Nutrition 49, no. 2: 91–99. 10.1007/S00394-009-0053-7. 19760359

[nhs70000-bib-0047] Zhang, Q. , S. Y. Chair , S. H. S. Lo , J. P. C. Chau , M. Schwade , and X. Zhao . 2020. “Association Between Shift Work and Obesity Among Nurses: A Systematic Review and Meta‐Analysis.” International Journal of Nursing Studies 112: 103757. 10.1016/J.IJNURSTU.2020.103757. 32921429

[nhs70000-bib-0048] Zhang, Y. , J. Murphy , H. M. Lammers‐van der Holst , L. K. Barger , Y. J. Lai , and J. F. Duffy . 2023. “Interventions to Improve the Sleep of Nurses: A Systematic Review.” Research in Nursing & Health 46, no. 5: 462–484. 10.1002/NUR.22337. 37710916 PMC10539041

