# Supplementary material for: Balancing Knowledge and Health: A Comparative Analysis of Students and Healthcare Workers Nutrition Related Health Behaviors, a Cross‐Sectional Study
Source: Nurs Health Sci. 2024 Nov 18;26(4):e70000. doi: 10.1111/nhs.70000 (PMC11586509; doi:10.1111/nhs.70000)
Supplement: Supplementary file 1 — Data S1. [file NHS-26-e70000-s001.docx]

Instruction: For question numbers 1-20 select one of the following options as your response: (a) Significantly increased (b) Slightly increased (c) Grossly similar (d) Slightly decreased (e) Significantly decreased.

From the start of your studies period/ from the beginning of the period of your work profession:

1. How has your probability of skipping one of the main meals (breakfast/lunch/dinner) changed?
2. How has your habit of snacking between meals changed?
3. How has your quantity/portions of meals and snacks changed?
4. How has your daily intake of fruits and vegetables changed?
5. How has your intake of a balanced diet (including healthy ingredients such as whole wheat, pulses, legumes, eggs, nuts, fruits and vegetables) changed?
6. How has your consumption of junk food/fast food and fried food changed?
7. How your intake of sugar-sweetened beverages (carbonated soft drinks, sugar-sweetened juices) changed?
8. How has your consumption of sweets/candies/chocolate changed?
9. How has your participation in cooking new/traditional recipes changed?
10. How has your consumption of unhealthy food when you are bored or stressed or upset changed?
11. How has your intake of immunity-boosting foods (lemon, turmeric, garlic, citrus fruits and green leafy vegetables) in the diet changed?
12. How has your intake of nutrition supplements to boost immunity changed?
13. How has the support of your family and friends in eating healthy changed?
14. How has your interest in learning healthy eating tips from the media (newspaper articles/magazines blogs/videos/TV shows/text messages) changed?
15. How has your participation in aerobic exercise changed?
16. How has your participation in leisure and household chores changed?
17. How has your sitting and screen time changed?
18. How have your hours of sleep changed?
19. How has your quality of sleep changed?
20. How have your stress and anxiety levels changed?
